# Selectable Implant Removal Methods due to Mechanical and Biological Failures

**DOI:** 10.1155/2017/9640517

**Published:** 2017-07-05

**Authors:** Jong-Bin Lee

**Affiliations:** ^1^Department of Periodontology, School of Medicine, Ewha Womans University, Seoul, Republic of Korea; ^2^Department of Periodontology, Research Institute for Periodontal Regeneration, College of Dentistry, Yonsei University, Seoul, Republic of Korea

## Abstract

Dental implant has been restoring the function and esthetics lost from missing tooth. However, biomechanical implant complications are the major cause of failing implants. Therefore, implant removal is one of the indispensable dental treatments. The 70-year-old male and 66-year-old female who had discomfort on posterior implants region came to Department of Periodontology. Conventional method using trephine bur and the new, nontraumatic method using a fixture removal kit were used for implant removal, respectively. Two different methods are commonly used for implant removal. Each has advantages and disadvantages; thus, the applied surgical method must consider a patient's intraoral condition, posttreatment plan, and the level of surgeon's skill and experience. In conclusion, strategically executing the most optimal implant removal method plays a pivotal role in maximizing the success rate of implant reinstallation that follows afterwards.

## 1. Introduction

Dental implant is a common treatment for edentulous region. Many studies have proven the success of the dental implants. Guided bone regeneration (GBR) is followed in the cases of implants on poor residual alveolar bone conditions. Even so, over 90% success and survival rate has been reported [1, 2].

Occurrences of concomitant complications are on the rise due to increased number of patients seeking dental implant installation. An in-depth study is warranted in preparation for combating rising complications. Implant complications can be divided into two major categories: mechanical and biological complications. Mechanical complications include loosening or fracture of abutment screw, damage or fractures of fixture or abutment, and fracture or fall-out of prosthodontics. These cause unwanted stress on implant and the tissues around it and may trigger additional complications. Biological complications include peri-implant mucositis and peri-implantitis [3]. These develop a bone resorption and failing osseointegration; thus inflammation and pain can also arise. Such complications may lead to failing implant treatment [4, 5].

Fracture of an implant fixture or peri-implantitis aggravates surrounding tissue damage and causes a failing implant; thus implant reinstallation should be planned after explanting implant fixture and enough healing phase [6–8].

Accordingly, the study about implant removal is very important as the preceding step of reimplantation. In previous study, various implant removal methods using different tools, such as scalpel instrument, bur and forceps only, bur, forceps, and elevator, trephine drill, and implant removal kit, have been introduced [9].

The presence of natural tooth and another implant adjacent to the failing implant and the thickness of buccal and palatal/lingual cortical bone must be considered in selection of implant removal method [9]. When the failing implant is neighboring natural tooth or another implant, the use of fixture removal kit would be a safe option. Also, previous studies reported that conservative method using fixture removal kit should be preferentially selected in all cases that implant removal is required in. But, trephine bur can be used when the thickness of cortical bone around the implant is more than 1.5 mm.

This case report examines two failing implant cases due to both mechanical and biological complications and presents two different implant removal methods that can be applied in such cases.

## 2. Case Presentation

### 2.1. Case  1

#### 2.1.1. Presurgical Examination

**Figure 1:**
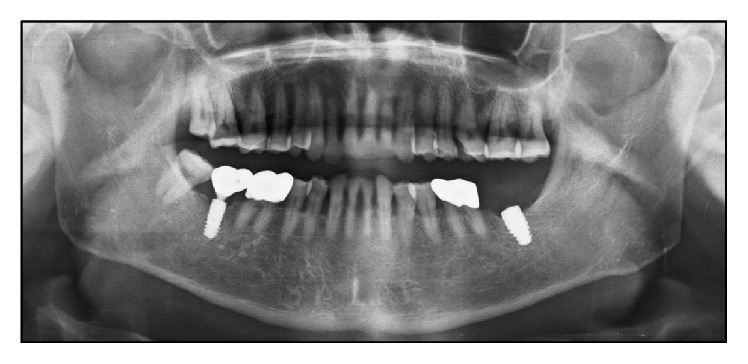
Preoperative radiographic view (panoramic X-ray image). Implant of #37 area shows the broken abutment screw off the implant fixture inside.

A 70-year-old male requested Department of Periodontology to remove the implant fixture at second molar region of left mandible (Figure 1). The patient stated that a local clinic had attempted and failed to remove the broken abutment screw off the implant fixture inside. Furthermore, the implant fixture was instead damaged and fractured in the previous process. The patient was then referred to this hospital for the implant removal. Traces of round bur usage for screw removal, serious damage on upper part of the fixture, and gingival defect were observed. The patient was diagnosed with broken abutment screw through clinical and radiographic examination inside the implant fixture, and an implant removal was planned with the patient's consent.

#### 2.1.2. Surgical Procedures

**Figure 2:**
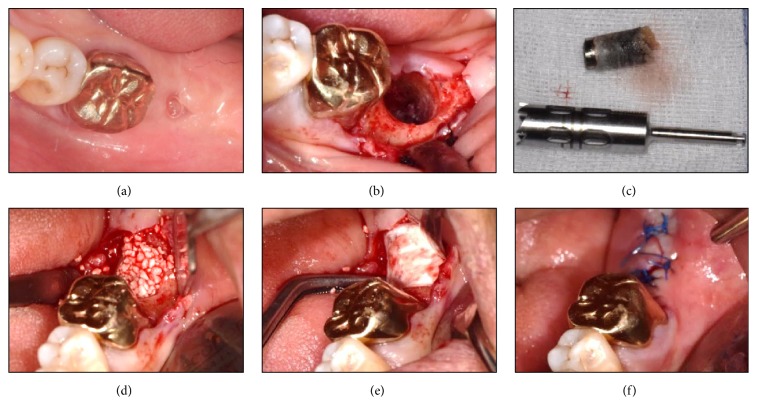
Conventional removal method applied on #37 region. (a) Preoperative state showing a gingival defect. (b) Implant removal using a trephine bur (external diameter: 6 mm, internal diameter: 5 mm; Biomet, Warsaw, IN, USA), elevator (Hu-Friedy, Chicago, IL, USA), and root forceps (Hu-Friedy, Chicago, IL, USA). (c) The implant and the surrounding alveolar bone removal. ((d) and (e)) Immediate bone grafting [Osteon II 0.5 g (particle size 0.5~1.0 mm) and collagen membrane 15 × 20 mm; Genoss, Suwon, Korea] on the implant removal socket. (f) Suture for a primary wound closure.

Block and infiltration anesthesia were administered on the surgical site. Upon anesthesia, intracrevicular and crestal incisions were followed using #12 and #15 blades. A clear line of sight of the exposed implant was secured by the full thickness flap elevation on the alveolar ridge crest. Trephine bur (Biomet, Warsaw, IN, USA) with 6 mm outer diameter and 5 mm inner diameter was placed on top of the implant fixture to make sure that it fits inside the bur. The depth of the implant fixture was measured on panoramic X-ray image, and its inclination was compared to the adjacent teeth. Based on these, the implant fixture and surrounding bone were drilled using trephine bur at low speeds under saline irrigation. The loosen implant fixture was removed by a dental elevator (Hu-Friedy, Chicago, IL, USA) and root forceps (Hu-Friedy, Chicago, IL, USA) with care in order not to damage the alveolar bone. For the implant reinstallation, the guided bone regeneration using Osteon II 0.5 g (particle size 0.5~1.0 mm; Genoss, Suwon, Korea) and 10 × 20 mm collagen membrane (Genoss, Suwon, Korea) was applied on the widely formed implant removal socket. After placing buccal and lingual flap properly, interrupted suture was given on the spot for primary closure (Figure 2).

#### 2.1.3. Follow-Up Examination

**Figure 3:**
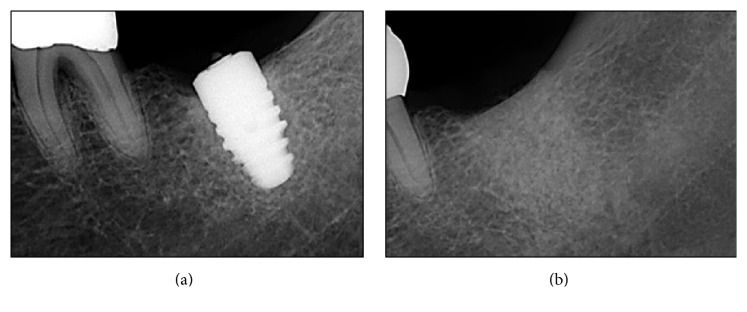
Postoperative radiographic view (periapical X-ray image). (a) Implant fixture with a fractured abutment screw. (b) Implant removal site filled with a bone grafting material.

**Figure 4:**
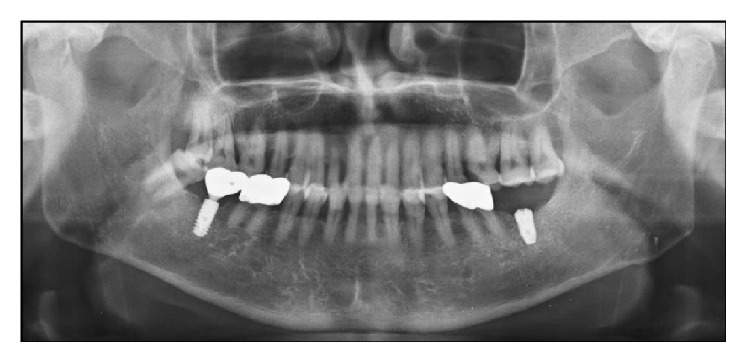
Postoperative radiographic view (panoramic X-ray image). Implant reinstallation on #37 region four months after the implant removal.

Periapical X-ray image verified the complete implant removal after the surgery (Figure 3). Two weeks after the surgery, the patient showed normal healing phase as the stitches were removed. Two months after the surgery, completely cured soft tissues of the surgical site were verified; thus reinstallation of an implant was planned and carried out four months later (Figure 4).

### 2.2. Case  2

#### 2.2.1. Presurgical Examination

**Figure 5:**
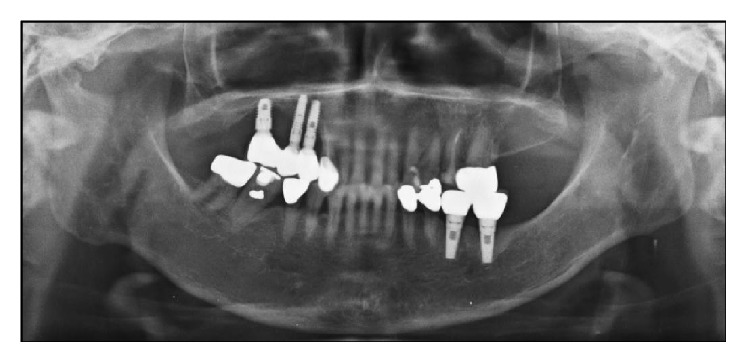
Preoperative radiographic view (panoramic X-ray image). Implants of right maxilla area show a severe alveolar bone loss lesion.

A 66-year-old female who had discomfort on implants of first premolar, second premolar, and first molar region of right maxilla came to Department of Periodontology (Figure 5). Those were treated at a local clinic seven years prior to this visit. She experienced discomfort due to recurring implant prosthodontics falling-out. Peri-implantitis derived from the failing implants gave her frequent pain. The radiographic examination verified the radio-lucency lesion around the implant fixtures, and gingival recession was observed in the clinical examination. Upon the patient's strong demand of removing the implants and consent, surgical procedure was scheduled.

#### 2.2.2. Surgical Procedures

**Figure 6:**
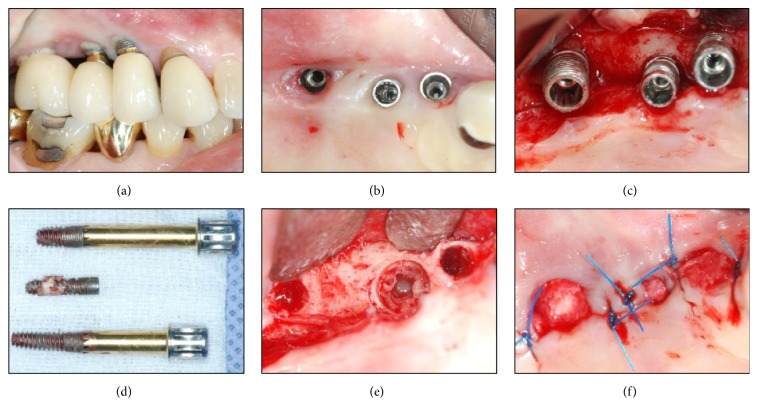
Advanced and mixed removal methods applied on #14, #15, and #16 regions. (a) Supragingival eruption state of an implant fixture. (b) Implant prosthodontics removal state showing a lack of keratinized gingiva and peri-implant tissue impairment. (c) Exposure of upper portion of the implant fixture and severe surrounding alveolar bone loss. (d) Implant removal using the Neo FR Kit (NeoBiotech, Seoul, Korea) on #14 and #16 regions and using the trephine bur (external diameter: 5 mm, internal diameter: 4 mm) on #15 region and the surrounding alveolar bone. (e) Implant removal socket showing a perforation of the sinus inferior wall and maintenance of the internal schneiderian membrane. (f) Tension-free suture for a natural healing progress.

Block and infiltration anesthesia were administered on the surgical site. Intracrevicular and crestal incisions were performed simultaneously using #12 and #15 blades, and a clear line of sight of the exposed implant was secured by elevating the full thickness flap. Afterwards, Neo FR Kit (NeoBiotech, Seoul, Korea) was used for explantation. The fixture removal screw and driver were placed inside the implant fixtures at first premolar and first molar regions. Subsequently, sufficient amount of force was exerted using a hand wrench in the opposite direction of the installed implants. The implants became loose enough to be removed. The Neo FR Kit was also used to try to remove the implant at second premolar region but failed to do so. Therefore, after the depth and inclination of the implant were analyzed through panoramic X-ray image, the implant and the surrounding bone were drilled under saline irrigation by trephine bur with outer diameter of 6 mm and inner diameter of 5 mm at low speeds. A perforation of inferior alveolar bone of the right sinus was discovered during the procedure but the schneiderian membrane of internal space was still preserved and not torn. Thereafter, the implant was loosened by an elevator and extracted by root forceps. Interrupted suture was given on the surgical site for primary closure. Immediate implant reinstallation was impossible on the same site because of the alveolar bone loss and a need for inflammation treatment (Figure 6).

#### 2.2.3. Follow-Up Examination

**Figure 7:**
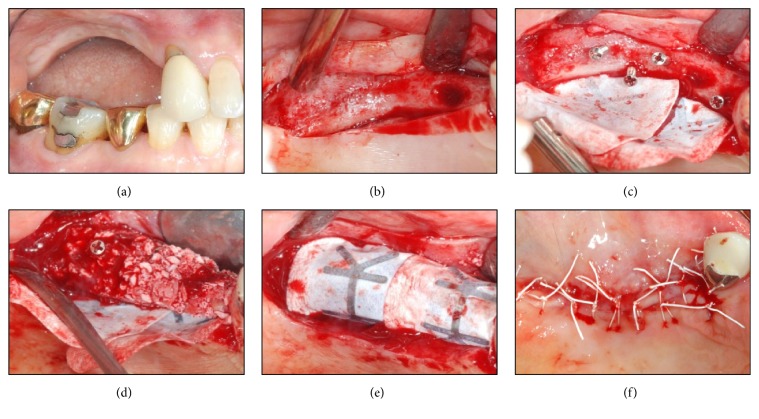
Vertical augmentation of alveolar ridge applied on the implant removal site. (a) Severe vertical resorption state four months after the implant removal. (b) Narrow alveolar ridge. (c) Insertion of a tenting screw (Dentium, Seoul, Korea) and application of a GORE-TEX membrane TR6Y (W. L. Gore & Associates Inc., Flagstaff, Arizona, USA). ((d) and (e)) Bone grafting using a Bio-Oss 1.0 g (Geistlich Pharma AG, Wolhusen, Switzerland), and GORE-TEX membrane covering the site and supported by the tenting screw. (f) Suture for a primary wound closure.

**Figure 8:**
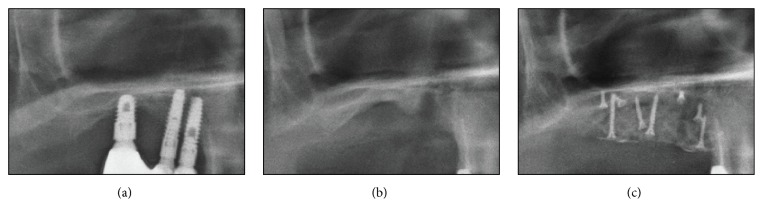
Postoperative radiographic view (periapical X-ray image). (a) Implant fixture with severe surrounding alveolar bone loss. (b) Implant removal site. (c) Vertical augmentation of an alveolar ridge.

**Figure 9:**
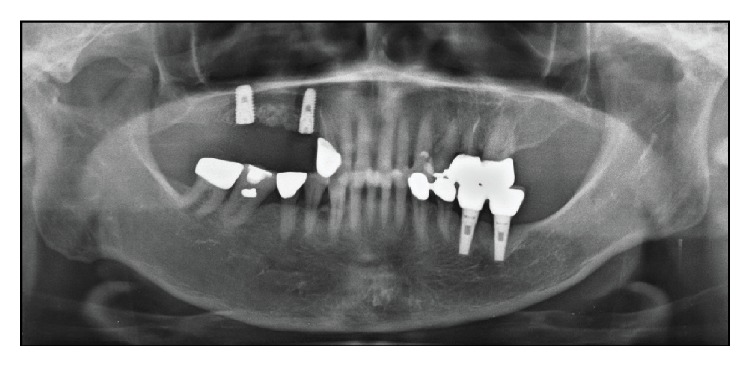
Postoperative radiographic view (panoramic X-ray image). Implant reinstallation on #14 and #16 regions four months after the implant removal.

Panoramic X-ray image verified successful implants removal (Figures 8(a) and 8(b)). Stitches were removed two weeks after the surgery, and normal healing phase was observed four months after the surgery. The vertical alveolar ridge augmentation using Bio-Oss 1.0 g (Geistlich Pharma AG, Wolhusen, Switzerland) and GORE-TEX membrane TR6Y (W. L. Gore & Associates Inc., Flagstaff, Arizona, USA) was conducted on the implants' removal regions. These sites were supported by tenting screws (Dentium, Seoul, Korea) to secure vertical height (Figures 7 and 8(c)). Four months after this treatment, two implants were reinstalled at first premolar and first molar regions (Figure 9).

## 3. Discussion

In cases of implant failure due to biomechanical complications, various implant removal methods may apply [10]: (1) trephine bur, (2) thin bur at low speed under saline irrigation, (3) electrosurgery (i.e., thermal explant), and (4) fixture removal kit. Methods (1), (2), and (3) are considered conventional methods, whereas (4) is the latest and most sophisticated method. Conventional method was applied in the first case, and both conventional and latest methods were used in the second case.

The downside of the conventional method is causing damage on alveolar bone surrounding the implant, so a bigger diameter implant is necessary for an immediate reinstallation [8, 10, 11]. In contrast, a fixture removal kit helps preserving the size of the implant removal site; thus immediate reinstallation is possible. However, any screw or abutment fragments may obstruct placing the instrument inside a screw hole; thus using a removal kit becomes difficult [12].

If an implant is cut and then separated by a diamond bur placed inside the implant fixture, compromised surrounding bone can be minimized after the implant removal. Consequently, an immediate implant reinstallation achieved a successful result [13].

In other words, conventional method and advanced method using fixture removal kit each have advantages and disadvantages. However, the latter minimizes the damage of tissues around the implant and facilitates reimplantation; thus it can be the first choice of treatment method for clinician. It can also be a useful treatment method in terms of reduction in treatment time and noise and having less stress on both dentists and patients [9].

The conventional method, which was used in the first case, is the most common and relatively simple in terms of tools use. It only requires a trephine bur and extraction instruments [14–16]. Therefore, it will be more appropriate choice when fractured implant fixture or abutment screw needs to be removed or when an implant fixture is too integrated into the bone. However, the compromised surrounding alveolar bone is unavoidable from the conventional method, so either implant fixture with bigger diameter or bone grafting treatment must be accompanied. That is, implant reinstallation on the same site becomes more complicated after the conventional method is applied [10].

Fixture removal kit was used in order to remove the implant fixture at first premolar and first molar regions in the second case. After placing fixture removal driver inside the implant fixture, torque was applied in the opposite direction of the installed implant fixture to loosen it. This method minimizes surrounding alveolar bone damage; thus a new implant with the same diameter can be immediately reinstalled on the same site [10]. On the other hand, when excessively developed osseointegration presents as in the first case and at second premolar region in the second case, the fixture removal kit is less preferred due to excessive torque exertion. Since the excessive torque creates too much stress on the alveolar bone, it may lead to compromised surrounding alveolar bone and damage in inner structure of the implant fixture [16]. Therefore, conventional method seems like a more rational choice in such a case [14, 15].

The implant removals were successful in both cases, and the patients' postoperation condition was also stable. Future implant reinstallation was therefore scheduled.

For a more detailed reporting of implant removal methods, a number of successful clinical cases done by various methods and follow-up treatment including implant reinstallation would be required. If drilling alveolar bone must be involved in an implant removal process, it must be conducted under saline irrigation with care in order to minimize surrounding alveolar bone damage. Also, alveolar bone loss around the treatment site must be minimized by using a trephine bur that has similar diameter to fixture, and GBR should be considered in fixture removal socket in order to allow reimplantation later. In case of using a commercially available fixture removal kit, the higher success rate would be expected with a clinician's enough knowledge of how to handle the kit. Otherwise, there might be issues such as fracture of kit's instrument and fixture due to excessive fixture removal torque application, or increase of stress on tissue around treatment site. Also, patient's intraoral conditions observed during the implant removal should be considered in determining the timing and viability of implant reinstallation procedure.

Since both patients in this case report were the elderly, consideration of systemic diseases, which should be taken care of in the normal course of surgery, is essential [17, 18]. Therefore, establishing clear future treatment plan is also important. Selection of implant removal method and application of GBR can be shifted depending on either reimplantation or exchange of removable partial denture (RPD). GBR is integral in reimplantation; and the ridge preservation procedure should be taken into account for ensuring RPD retention.

Reimplantation was performed after 6 months and 8 months in this study, respectively. Healing period of one year or more is not necessary after implant removal, and soft and hard tissues were healed enough for reimplantation after 9 months [19, 20]. Easier reimplantation would have been expected in our case if an additional healing period of 1 to 3 months had been secured.

## 4. Conclusion

Implant failures due to biomechanical complications ultimately require implant removal; hence various methods for implant removal have been attempted and reported. If a dentist is to select removal method before operation, total treatment plan, the patient's intraoral condition, and the level of surgeon's skill and experience must be taken into consideration. Implant removal is in close association with implant reinstallation. Therefore, proper implant removal method needs to be selected after careful treatment planning is discussed with the patient. For immediate reinstallation of an implant, more conservative approaches should be considered.
